# Exploring the effects of riverine flooding on traffic demand forecasting using activity-based modeling in Ubon Ratchathani, Thailand

**DOI:** 10.1038/s41598-026-42434-x

**Published:** 2026-03-19

**Authors:** Noriyasu Tsumita, Rattanaporn Kaewkluengklom, Sideney Schreiner, Sittha Jaensirisak, Atsushi Fukuda

**Affiliations:** 1 Faculty of Transdisciplinary Sciences for Innovation, Kanazawa University, Kanazawa, 920-1192 Japan; 2 Faculty of Civil Engineering, Ubon Ratchathani University, Ubon Ratchathani, 34001 Thailand; 3 PTV Group Japan, Tokyo, 1560057 Japan; 4 College of Science and Technology, Nihon University, Chiba, 274-8501 Japan

**Keywords:** Activity-based model, Riverine flooding, Activity, Traffic demand forecasting, Adaptation measure, Climate sciences, Environmental studies, Geography, Geography, Natural hazards, Social policy

## Abstract

Riverine floods are a recurring and increasingly severe problem in Southeast Asia, often resulting in the submersion of large urban areas for extended periods. As a result, urban mobility is severely disrupted, significantly affecting the daily activities of residents. Integrating behavioral changes during floods into travel demand forecasting is essential for assessing flood adaptation measures in the transportation sector. However, no detailed analysis has been conducted on the impacts of urban flooding on daily activities and travel behavior, and no examples are available on how to incorporate these impacts into travel demand forecasts. This study proposes the development of an activity-based traffic demand forecasting model that explicitly incorporates behavioral changes in individual activity patterns during floods. The model is designed to reflect the disruptions caused by riverine floods and their impact on daily travel behaviors, road closures, and reduced accessibility, which are often overlooked in traditional forecasting methods. Then, the model evaluates adaptation measures in the transportation sector (such as the elevation of arterial roads), supporting strategic planning for resilient urban mobility during floods.

## Introduction

In recent years, riverine floods have been a recurrent problem in Southeast Asian cities; their intensity and extent of damages caused have escalated over time^1–3^. The insufficient amount of leaves in many rivers has resulted in extensive floods in urban areas. In addition, most cities are located in low-lying regions with no elevation differences, resulting in poor drainage and floods that last over a month, causing substantial damage to properties, buildings, and infrastructure; additionally, road network connectivity is disrupted for extended periods and urban mobility is reduced, significantly affecting the daily activities of residents^4^.

Traditionally, even in the absence of formal land-use regulations, flood-prone areas were typically avoided for residential and urban development due to long-standing local knowledge of their hazard potential. Water-retention basins, stilt houses, and small boats have been employed as flood adaptation measures^5,6^. As urban development is accelerating, areas with high flood risk and normal water-retention facilities have undergone extensive development, leading to urbanization and motorized transportation, including passenger cars and motorcycles^7^. Consequently, structural flood mitigation measures, such as floodwalls and dams, have been implemented in Thailand and other countries^8^. Nonetheless, the lack of dams in many rivers has resulted in large-scale floods recurring, causing significant damage. During floods, residents often adapt to their daily activities; however, urban mobility is substantially reduced.

Despite implementing measures against global warming, extreme weather phenomena causing floods are expected to increase. Consequently, the existing flood-control measures are insufficient to prevent flood-related damage, emphasizing the need for adaptation measures. During floods, such measures must ensure a certain level of urban mobility, which is essential for maintaining the overall urban function. Therefore, adaptation measures should be incorporated into transportation planning. This can be achieved by understanding the changes in human activities during extended flood periods and incorporating the findings into traffic demand forecasting to assess the effectiveness of the adaptation measures.

However, previous studies have not investigated such changes during riverine floods. Therefore, it is crucial to evaluate and implement adaptation measures in the transport sector that account for these changes in traffic demand forecasting. By this, developing resilient transportation systems and networks that ensure a certain level of mobility even during floods is important to maintain daily activities and travel behaviors as usual.

Furthermore, previous studies for evaluating the effectiveness of adaptation measures in the transportation sector have generally relied on conventional four-step traffic demand model (four-step model). However, during flood events, people’s daily activities change significantly, and it is therefore essential to explicitly incorporate these behavioral changes into activity-based travel demand forecasting.

Thus, the objective of this study is to clarify how long-period riverine flooding affects traffic demand and individual activity patterns using an Activity-Based Model (ABM).The specific research questions are as follows:

1. How do individuals change their activity patterns in order to adapt to riverine flooding?
2. How can a traffic demand forecasting method be developed using ABM based on survey results?
3. How effective are adaptation measures in maintaining urban mobility in the transport sector during riverine floods, as evaluated using an ABM?

This study is organized as follows. Section “Literature review” reviews previous studies on the analysis of activity and travel behavior, as well as methods for evaluating adaptation measures in the transport sector during riverine floods. Section “Methodology” presents the methodology for traffic demand forecasting using ABM, together with calibration results based on observed traffic data under normal conditions. Section “Adaptation measures in the transport sector and scenarios” describes the method for prioritizing adaptation measures and defining scenarios. Section “Results of the case study” presents the traffic demand forecasting results for each scenario and evaluates the adaptation measures using multiple indices. Finally, Section “Conclusion” concludes the study.

## Literature review

The behavioral changes caused by urban floods have been investigated in previous studies. Trip-based travel behaviors focusing on changes in individual trips during floods have been reported in some previous studies. An empirical study on the impact of climate change and weather conditions on the transport sector clarifying the differences in the changes in modal choice due to different weather conditions has been reported in the past study^9^. Questionnaire-based surveys on people’s travel behaviors during floods and other natural disasters have been reported in some studies^10–12^. More recent studies have further expanded this line of research by examining mobility responses to extreme precipitation and flood events using empirical and large-scale data. For example, Ye et al. (2025) analyzed urban mobility responses to extreme rainfall events in Zhengzhou, China, and demonstrated significant reductions and temporal shifts in travel demand under severe precipitation conditions^13^. Similarly, Yao et al. (2024) investigated automobile commuters in Shanghai and found that flooding under climate change scenarios substantially alters commuting patterns, including trip frequency and departure time adjustments^14^. Talpur et al. (2025) examined travel decision-making during extreme floods in Pakistan and highlighted behavioral adaptations such as trip cancellation, route changes, and mode substitution under high-risk conditions^15^.

Conversely, transportation has been regarded as a derivative of activities. Changes in activities during floods have been investigated by conducting questionnaire-based surveys, focusing on the individual relationships between travel behaviors and activities. For example, Liu et al. (2014, 2020) investigated such activities under different weather conditions by analyzing national traffic survey data and applying simultaneous-equation Tobit models in Sweden^16,17^. They found a tradeoff relationship between routine and extraordinary activities under unusual weather conditions. Rudloff et al. (2015) clarified the effects of weather on activity using long-term household traffic surveys^18^. They applied a statistical model based on these results, showing that weather significantly influences people’s modal choices and activities depending on personal attributes. Cools et al. (2010, 2013) conducted a questionnaire-based survey to investigate the impact of weather information (including floods) on activities and travel behaviors^19,20^. They found that weather conditions information significantly affect the modal choice and the departure times.

In numerous studies, traffic demand forecasting during floods has relied on the four-step models. This is a well-established and widely used method because of its effectiveness in trip-based traffic demand forecasting, particularly in assessing the impact of floods on urban mobility. Vajjarapu et al. (2020, 2021) developed a four step model that incorporates the impact of reduced number of trips and road network performance on urban mobility^21,22^. They found that the number of trips caused by the degradation of the road network service is reduced according to the flood depth. Chang et al. (2010) investigated the impact of urban floods on transportation infrastructure by evaluating the reduction in free flow speeds and the disruptions caused by floods to estimate traffic demand in current and future scenarios^23^. Furthermore, four-step models have been employed to estimate the degradation of road network service and the disruptions caused by floods and analyze their impact on urban mobility; additionally, they indicate the changes in trip distribution and modal choice^24,25^. However, they exhibit notable limitations. For example, they cannot accurately predict the reduction in the total number of trips caused by the degradation of the road network service; additionally, they cannot explain the mechanisms according to which daily activities and activity patterns change in response to flooding, particularly when considering the changes in socioeconomic characteristics and household compositions. More recently, Batur et al. (2024) extended this activity-based perspective by examining how extreme heat affects human activity–mobility interactions and daily time-use patterns, demonstrating that extreme climate conditions fundamentally reshape activity schedules as well as travel behavior^26^. In addition, Long and Duan (2025) emphasized that human mobility itself can amplify compound flood risks in coastal urban areas under climate change, highlighting the feedback mechanisms between activity–mobility patterns and flood exposure^27^.

In contrast, ABMs estimate travel demand by modeling the interdependence between activity schedules and travel behaviors. Therefore, they provide a more comprehensive understanding of how individuals reorganize their daily lives in response to external disruptions than four step models. Additionally, they improve the analysis of behavioral mechanisms by representing the changes in human activities as well as the choice of location and TM (Traffic Mode). Based on their advantages, ABMs have been employed in the evaluation of the adaptation measures for natural disasters and the assessment of transportation policies as alternatives to four-step models.

However, only a few studies have employed ABMs in the evaluation of the impact of urban floods on travel behaviors and traffic conditions. Pyatkova et al. (2019) employed an ABM to investigate the impact of flash floods on traffic conditions by modeling the reductions in free flow speeds across individual road segments^28^. Yu et al. (2022) forecast future travel demand and assessed various adaptation measures for coastal floods caused by typhoons in Miami-Dade County, Florida^29^. Using vehicle hours traveled as a performance metric, they demonstrated that the impact of floods on traffic varies significantly, depending on the extent of flooding and the nature of human activities. Saadi et al. (2017) analyzed the impact of different magnitudes of riverine floods on road networks by evaluating the degradation of the road network service relative to usual conditions. Their findings showed a substantial decrease in activities, which in turn, affected the overall travel demand^30^.

Despite the above-mentioned studies, travel behaviors and activity patterns during long-term urban floods in Southeast Asian cities have not been investigated. Riverine floods lasting for extended periods force people to change their activities according to the flood conditions. Therefore, it is necessary to investigate the changes in individual activities during extended flood periods and propose a methodology for estimating traffic demand that explicitly reflects the impact of such changes. To achieve this, we employ an ABM in an actual historic flood.

## Methodology

In this section, we present the methodology used to evaluate the effectiveness of adaptation measures in the transport sector during riverine floods. First, an Activity Diary Survey (ADS) was conducted to examine how individuals modify their activity patterns and travel behaviors in response to riverine flooding. Second, based on the questionnaire survey data, an activity-based traffic demand forecasting model was developed. Finally, the results of the activity-based model (ABM) were validated using observed traffic condition data and the survey data. The details of each methodological step are described in the following subsections.

### Traffic demand forecasting by activity-based model

Many ABMs have been developed in previous studies; they can be broadly classified into three categories: (1) utility-based probabilistic methods employing discrete choice models^31–33^, (2) rule-based methods^34–37^, and (3) hybrid models integrating both approaches^28–30^.

In this study, we employed the SIMBA MOBi model integrated into the PTV Visum software. It is a hybrid model developed by Scherr et al. (2020) for extracting the relationship between flood depth and travel speed reduction during floods and incorporating this relationship into the model^38^. Furthermore, the model identifies the behavioral changes caused by flood-related disruptions such as increased detour distances, changes in activities, start times of trips, and road closures.

These aspects are particularly distinctive features of this study because they enable a detailed and realistic representation of the urban mobility dynamics during floods. By integrating the dynamic characteristics of floods into the ABM, the changes in travel behavior and activity patterns during disruptions caused by floods can be effectively captured.

Although conventional ABMs were not designed to represent the impact of riverine floods on urban mobility, in this study, we extend the model to incorporate the flood-related effects on travel demand, thereby enabling the evaluation of adaptation measures in the transportation sector.

**Fig. 1:**
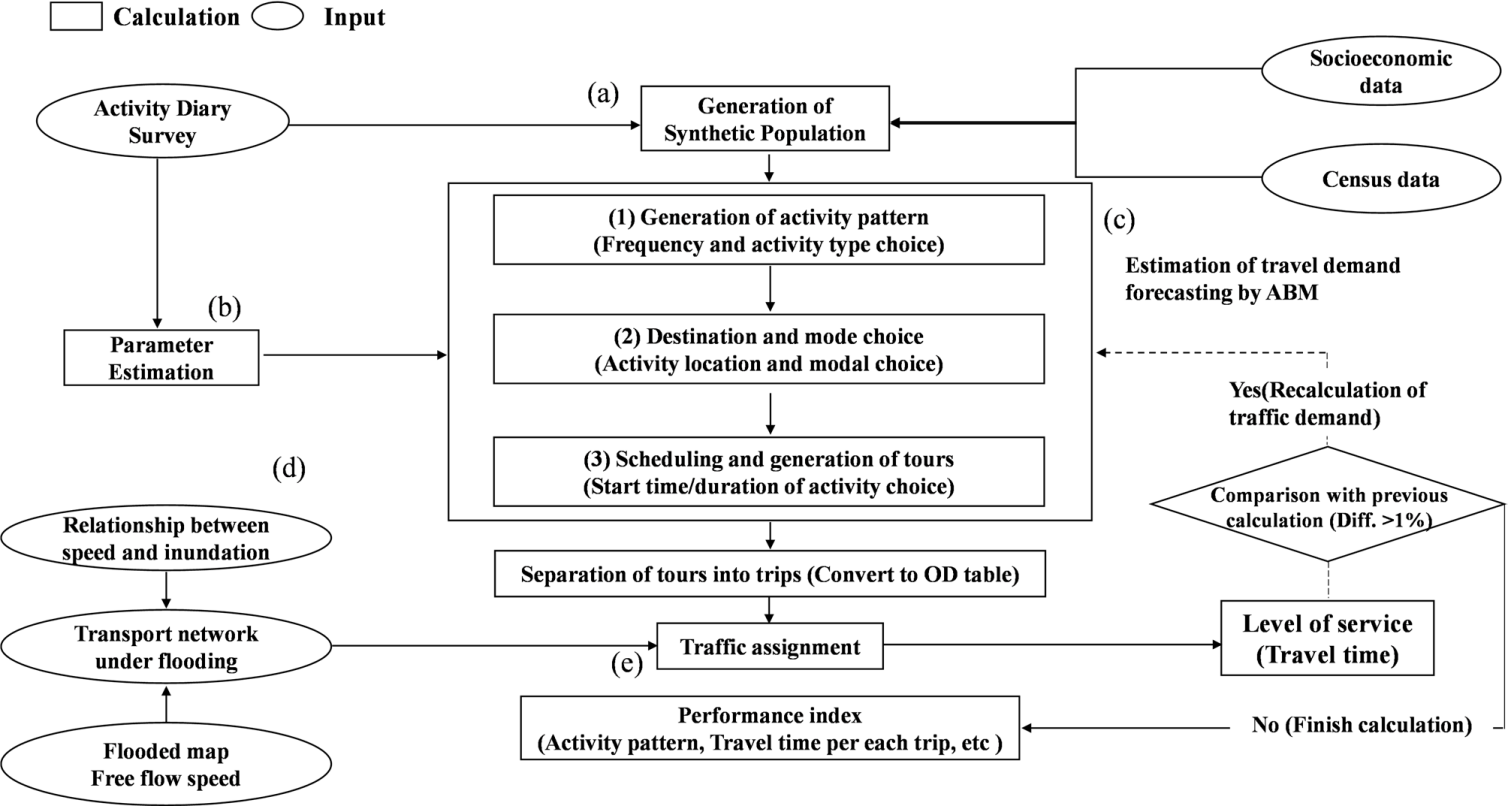
The framework of traffic demand forecasting by ABM.

The framework for traffic demand forecasting by ABM in this study is illustrated in Fig. 1 and includes five key steps: (a) generation of synthetic population data, (b) identification of changes in activities and estimation of the necessary parameters, (c) traffic demand forecasting based on ABM, (d) traffic assignment, and (e) representation of flood effects in the model.

#### Generation of synthetic population

ABM estimates traffic demand in urban areas by formulating the relationship between individual activities and travel behaviors to indicate the changes in personal and household characteristics during the day. For this purpose, disaggregated synthetic populations, which include such characteristics, are essential. This study employed the synthetic population interactive proportional updating (IPU) method to calculate the expansion factors and generate the synthetic population data^39–41^. These factors are calculated using the marginal frequencies obtained from multiple aggregate tables and used as constraints. The IPU method simultaneously addresses individual and household attributes, overcoming the limitations of the iterative proportional fitting method.

Using the IPU method, we integrated the aggregated statistical data with the 2015 Person Trip Survey (PTS) data to generate a synthetic population dataset, comprising 68,555 households and 257,113 individuals^42^. The synthetic population was calibrated using citywide marginal distributions, including person-level attributes (age and gender) and household-level attributes (household size and household income), derived from official statistical summaries. These marginals were applied as global control totals, such that the remaining counts in each category were progressively reduced as households and individuals were generated until the overall distributions matched the target values.

Although the marginal controls were enforced at the citywide level, population totals at the Traffic Analysis Zone (TAZ) level were treated as exogenous inputs. Specifically, the population by TAZ was provided in the zonal input data and used as a fixed constraint when assigning households and individuals to spatial units, thereby ensuring consistency with observed spatial population patterns.

This methodology preserves the odds ratios across population segments and maintains the marginal distribution of each characteristic obtained from the source data while simultaneously calculating the augmented coefficient for each segment (Eq. 1). To assess the goodness-of-fit of the synthetic population, we used the coefficient of determination (R^2^) to compare the generated and observed marginal distributions, particularly for age and household income.

1 $$\phi = \frac{{(\mathop p\nolimits_{{\mathop i\nolimits_{1} }} , \cdots ,\mathop p\nolimits_{{\mathop i\nolimits_{j} }} , \cdots ,\mathop p\nolimits_{{\mathop i\nolimits_{k} }} \cdots ,\mathop p\nolimits_{{\mathop i\nolimits_{m} }} )(\mathop p\nolimits_{{\mathop i\nolimits_{1} }} , \cdots ,\mathop p\nolimits_{{\mathop i\nolimits_{j} + \mathop c\nolimits_{1} }} , \cdots ,\mathop p\nolimits_{{\mathop i\nolimits_{k} + \mathop c\nolimits_{2} }} \cdots ,\mathop p\nolimits_{{\mathop i\nolimits_{m} }} )}}{{(\mathop p\nolimits_{{\mathop i\nolimits_{1} }} , \cdots ,\mathop p\nolimits_{{\mathop i\nolimits_{j} + \mathop c\nolimits_{1} }} , \cdots ,\mathop p\nolimits_{{\mathop i\nolimits_{k} }} \cdots ,\mathop p\nolimits_{{\mathop i\nolimits_{m} }} )(\mathop p\nolimits_{{\mathop i\nolimits_{1} }} , \cdots ,\mathop p\nolimits_{{\mathop i\nolimits_{j} }} , \cdots ,\mathop p\nolimits_{{\mathop i\nolimits_{k} + \mathop c\nolimits_{2} }} \cdots ,\mathop p\nolimits_{{\mathop i\nolimits_{m} }} )}}$$

where, ϕ Odds ratio, $$P_{{ij}}$$: Proportion of cell ij, $$C_{1} {{\& }}C_{2}$$: Positive intgers such that $$i_{j} + C_{1} \le n_{j}$$$$\& i_{k} + C_{2} \le n_{k}.$$

**Fig. 2:**
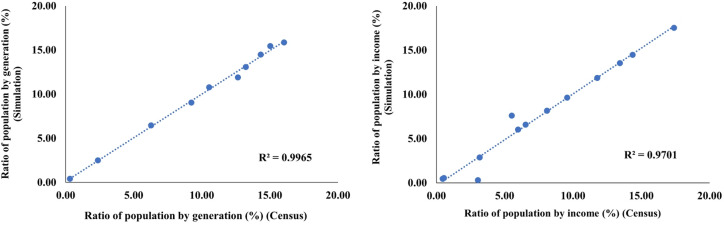
Distributions of the synthetic and actual populations according to age (left) and Income (right).

The synthetic population data were generated by performing repeated calculations and adjustments to the augmented coefficient for each element. To validate the accuracy of the generated data, this study compared the age and income distributions with the corresponding statistical data. Figure 2 shows that the synthetic population and actual statistical distributions are closely matched. The calculated coefficients of determination (R^2^) for the age and income distributions were 0.9965 and 0.9701, respectively, indicating high accuracy. The resulting R^2^ values exceeded 0.95 for these key attributes, indicating a high level of agreement between the synthetic and reference distributions and confirming the adequacy of the population synthesis results.

#### Identification of changes in activities and estimation of the necessary parameters

**Fig. 3:**
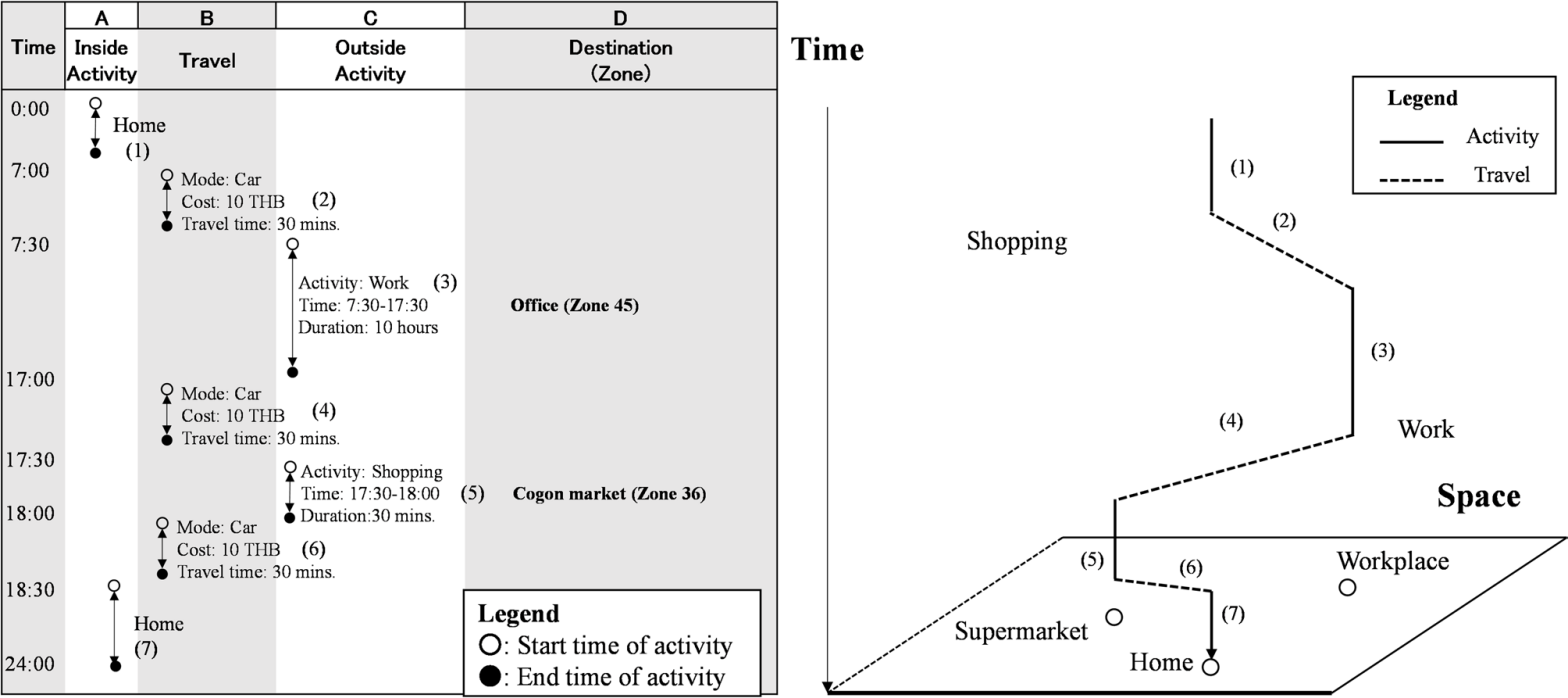
Sample of a completed ADS questionnaire.

This study conducted a face-to-face activity diary survey (ADS) in Ubon Ratchathani, Thailand, to assess the impact of floods on daily activities. Specifically, we collected detailed information about the respondents’ activities (e.g., start and end times, activity types, and locations) and travel behaviors (e.g., modes, travel times, and destinations) to analyze daily activity patterns and travel behaviors. The survey details are presented in Table 1. The survey included 300 households, comprising 810 individuals. The sociodemographic attributes obtained from the questionnaire survey, including age structure and annual income, were compared with the corresponding census data for the study area. The results indicate broadly similar trends between the two datasets. Specifically, the average age was 38.23 years old in the census and 34.76 years old in the survey, while the average annual income was 17,907 THB according to the census and 13,390 THB in the survey. These differences are considered modest, suggesting that the survey sample is reasonably representative of the target population. A sample of a completed ADS questionnaire is shown in Fig. 3.

**Table 1:** Detail of ADS.

Number of samples	810 participates(300 households)
Survey period	July–August 2022
Survey content (Individuals)	Travel behavior: (1) Destination, (2) Mode (3) Travel time (Departure and arrival time) (4) Route Activity: (1) Start and end time of activity (2) Content of activity, (3) Location of activity
Survey content (Households)	Household members of (1) Age (2) Income (3) Gender (4) Possession of car and/or motorcycle license (5) Employment status (6) Housing type (7) Vehicle ownership

**Fig. 4:**
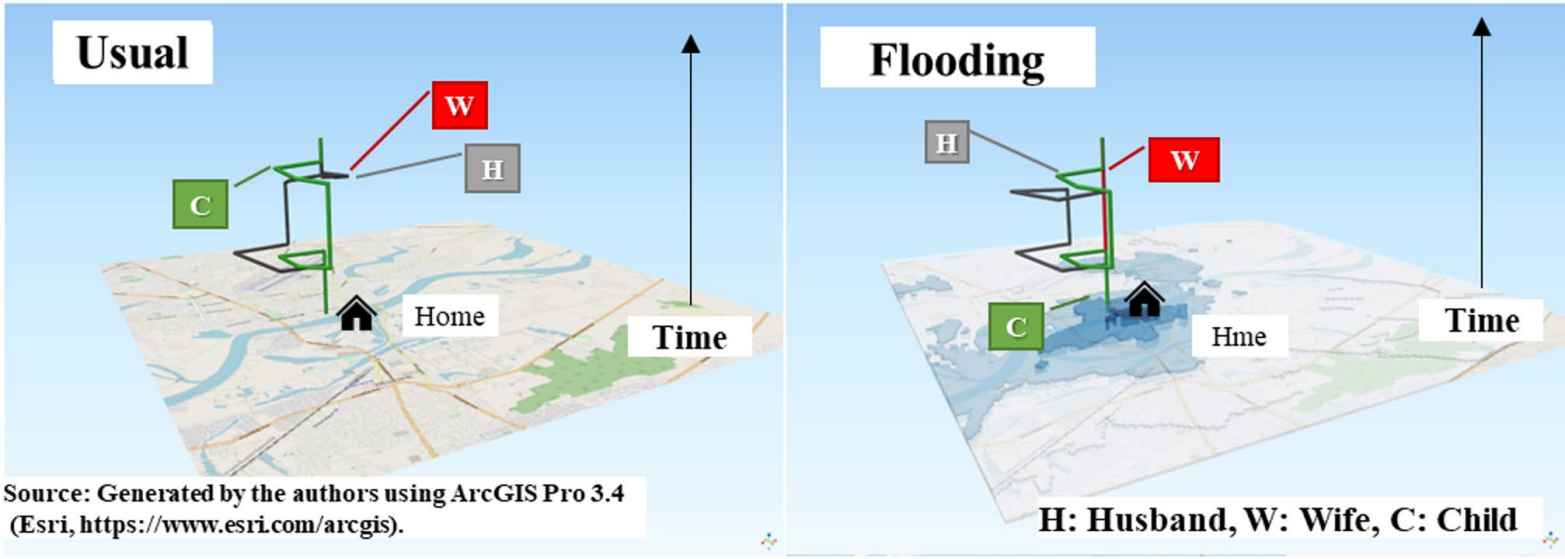
Spatiotemporal trajectories of a typical household (Left: Usual; Right: Flooding).

Based on the survey results, this study analyzed the spatiotemporal trajectories of household members affected by floods and represented the daily activity sequences as patterns (‟activity patterns.”) Fig. 4 illustrates the trajectories of household members (H: husband, W: wife, and C: child) under usual and flooding conditions, indicating the characteristic changes in activity patterns during floods. We observe that during floods, children’s activities remain essentially unchanged, wives stay at home throughout the day (canceling planned outdoor activities), and husbands alter their travel routes, making additional shopping stops on their way home.

The participants were categorized into three groups: workers, students, and nonworkers. The number of activity patterns in each group under usual and flooding conditions is presented in Table 2. Among workers, 34.7% commuted directly from home to work and back under usual conditions; however, figures decreased by approximately 10%–24.7% during flooding. In contrast, the proportion of workers who went shopping during work breaks increased by approximately 5.5% during flooding (usual conditions: 31.5%, flooding conditions: 37.0%), indicating a shopping tendency during work breaks or on the way home. Among students, approximately 70% commuted directly from home to school and back, indicating no significant difference between usual and flooding conditions. Among the nonworkers, 45.7% went shopping under usual conditions, dropping by approximately 10%–36.2% during flooding.

**Table 2:** Number of activity patterns in each group under usual and flooding conditions.

Classification	Activity pattern	Number of patterns (Usual)	Number of patterns (Flooding)	Difference
Worker	HWH	215 [34.7%]	153 [24.7%]	− 62 [− 10.0%]
	HWOH	81 [13.1%]	83 [13.4%]	2 [0.2%]
	HWSH	18 [2.9%]	21 [3.4%]	3 [0.5%]
	HWSWH	195 [31.5%]	229 [37.0%]	34 [5.5%]
Student	HEH	69 [71.9%]	64 [66.7%]	− 5 [− 5.2%]
	HOEH	16 [16.7%]	12 [12.5%]	− 4 [− 4.2%]
	HEOH	5 [5.2%]	5 [5.2%]	0 [0.0%]
Nonworker	HOH	9 [9.6%]	7 [7.5%]	− 2 [− 2.2%]
	HCH	7 [7.5%]	5 [5.3%]	− 2 [− 2.2%]
	HSH	43 [45.7%]	34 [36.2%]	− 9 [− 9.9%]
	H (staying all day)	147 [18.1%]	192 [23.6%]	45 [5.5%]

H: Home, O: Other, E: Education, S: Shopping. W: Work, C: Send children to school.

Using ABM, this estimated the logit model parameters, including frequency of tours and choice of activities (number of stops), and modal choice, based on data obtained from ADS. For the overall goodness of fit, the likelihood-ratio is presented to assess the power of the model with the estimated coefficient concerning the null model (where all coefficients are zero). For validation, a hit-ratio is presented to determine how accurate the model can predict the choice. Table 3 summarizes the estimated results regarding the frequency of tours among workers. In this study, a “Tour” variable indicates whether an individual conducted at least one out-of-home tour during the day. Specifically, “Without tour” represents individuals who did not go outside and stayed at home throughout the day, while “With tour” represents individuals who conducted at least one out-of-home tour. As no respondents in the questionnaire were observed to have conducted multiple tours within a single day, multiple tours were not considered in the analysis. Additionally, ASC represents the alternative-specific constant, and the values in parentheses indicate the t-values. In this study, t-values were summarized and discussed using significance levels of 10%: *(± 1.645), 5%: **(± 1.96), and 1%: ***(± 2.576). Assuming that tour occurrence is influenced by socioeconomic attributes and commuting distance, a binary logit model was estimated using an alternative-specific constant (ASC), age, distance to school or workplace, and ownership of a car or motorcycle as explanatory variables. The results show that the distance from home to the workplace/school and the availability of ownership of vehicles are statistically significant variables (significance levels: 1%). The calculated likelihood ratio was 0.414, indicating a reasonable fit for the model. Furthermore, the analysis confirms that an increase in the distance between home and workplace decreases the frequency of tours, indicating the significant impact of distance on travel behavior.

**Table 3:** Estimated result regarding the frequency of tours.

Variable	Without tour	With tour
ASC	–	− 1.651 (− 2.164**)
Age	2.505 (0.988)	− 2.507 (− 0.989)
Distance to workplace/school	0.017 (4.646)***	− 0.017 (− 2.646***)
Ownership of vehicle	–	0.766 (14.67***)
Likelihood	0.414	
Hit ratio	85.16%	

Table 4 presents the estimated results for the number of stop/drop activities conducted within daily activity patterns. Assuming that tour occurrence is influenced by socioeconomic attributes and commuting distance, a multinominal logit model was estimated using an ASC, age, the number of tours, distance to school or workplace as explanatory variables. The results indicate that the distance between home and workplace/school is a statistically significant determinant across all stop frequency categories, with significance at the 1% level. This suggests that commuting distance systematically influences individuals’ decisions regarding the number of intermediate stop/drop activities undertaken during the day. The variable “Age” and “the number of tour” also exhibit statistically significant effects for specific stop categories. In particular, age shows a positive and statistically significant association with the No. of Stop 2 category at the 5% significance level, while the number of tours is negatively associated with the “Two intermediate stops” category at the 10% significance level. These results imply that individuals with more complex daily tour structures tend to limit the number of additional stop/drop activities.

**Table 4:** Estimated result regarding the number of stop/drop activities.

Variable	Zero intermediate stop	One intermediate stop	Two intermediate stops
ASC	–	− 2.218 (− 2.394**)	− 2.544 (− 2.747***)
Age	− 0.474 (− 1.385)	0.371 (1.083)	0.077 (2.252**)
Number of tour	− 1.231 (− 0.545)	− 0.558 (− 0.635)	− 0.674 (− 1.745*)
Distance to workplace/school	− 1.036 (− 6.579***)	− 1.010 (− 6.417***)	− 1.037 (− 6.591***)
Likelihood	0.504		
Hit ratio	81.16%		

Although commuting distance consistently demonstrates strong statistical significance, the relatively small magnitude of the estimated coefficients suggests that its marginal effect on the choice of the number of stop/drop activities related to work and school is limited. Overall, the model achieves a likelihood ratio of 0.504 and a hit ratio of 81.16%, indicating a satisfactory level of explanatory power and predictive performance.

Table 5 summarizes the estimated results regarding the choice of stop/drop activities. Assuming that tour occurrence is influenced by socioeconomic attributes and commuting distance, a multinominal logit model was estimated using an ASC, age, distance to school or workplace, With or without children as explanatory variables. The ASC for the child pickup/drop-off alternative is relatively low and statistically significant at the 1% level, indicating that this activity is intrinsically less likely to be selected compared with the reference category, unless strongly influenced by individual or household attributes. This result highlights the importance of household composition in activity choice.The estimation results further indicate that the presence of children is a statistically significant determinant across all activity alternatives, with particularly strong effects on the selection of child pickup/drop-off and shopping activities. This suggests that household structure plays a critical role in shaping daily activity patterns beyond work and school commitments. Distance to the workplace or school also exhibits statistically significant effects. Specifically, the probability of selecting other activities decreases with increasing commuting distance, while longer distances are positively associated with child pickup/drop-off and shopping activities. This reflects behavioral adjustments in activity scheduling in response to spatial constraints. Regarding age, the results indicate that older individuals (excluding children) have a slightly higher likelihood of selecting child pickup/drop-off activities, although the effect on shopping activities is not statistically significant.

**Table 5:** Estimated result of parameter for activity choice.

Variable	Other	Chilren to send school	Shopping
ASC	–	− 6.747 (− 4.877***)	− 2.973 (− 1.400)
Age	0.061 (1.610)	0.915 (2.406**)	1.447 (0.381)
Distance to workplace/school	− 1.449 (− 3.456***)	1.599 (3.806***)	0.830 (1.979**)
With or without children	1.775 (2.955***)	1.411 (2.349**)	3.155 (5.252***)
Likelihood	0.185		
Hit ratio	65.54%		

Compared with other logit models estimated in this study, this activity choice model exhibits a lower likelihood ratio (0.185) and hit ratio (65.54%), suggesting relatively limited explanatory power. This outcome is expected, as work and school activities are predetermined in the synthetic population generation process, and destination choices are primarily governed by distance-based rules rather than utility-based optimization. These constraints limit the behavioral variability captured by the activity choice model and indicate potential areas for future refinement.

Table 6 summarizes the estimated results for the transport mode choice model. Assuming that tour occurrence is influenced by socioeconomic attributes and commuting distance, a multinominal logit model was estimated using an ASC and travel time as explanatory variables. The results indicate that the alternative-specific constants (ASCs) for car (driver), car (passenger), and motorcycle are positive and statistically significant at the 1% level, suggesting a strong inherent preference for private motorized modes relative to the reference alternative. In contrast, the ASC for public transport is not statistically significant, and walking is treated as the reference mode.

**Table 6:** Estimated result of parameter for modal choice.

Variable	Car	Car (passenger)	Motorcycle	Public transport	Walking
ASC	3.192 (12.15***)	2.006 (7.413***)	2.755 (10.33***)	− 0.233 (− 0.425)	–
Travel time	− 0.013 (− 0.425)	− 0.012 (− 0.485)	− 0.011 (− 0.342)	− 0.039 (− 1.149)	− 0.001 (− 0.328)
Likelihood	0.312				
Hit ratio	73.15%				

Travel time exhibits negative coefficients across all transport modes, indicating a disutility associated with longer travel times. However, none of the travel time coefficients are statistically significant at conventional significance levels. This outcome is primarily attributed to the limited variation and relatively small number of observations for public transport and walking modes in the survey data, which restricts the statistical power of the estimation.

**Fig. 5:**
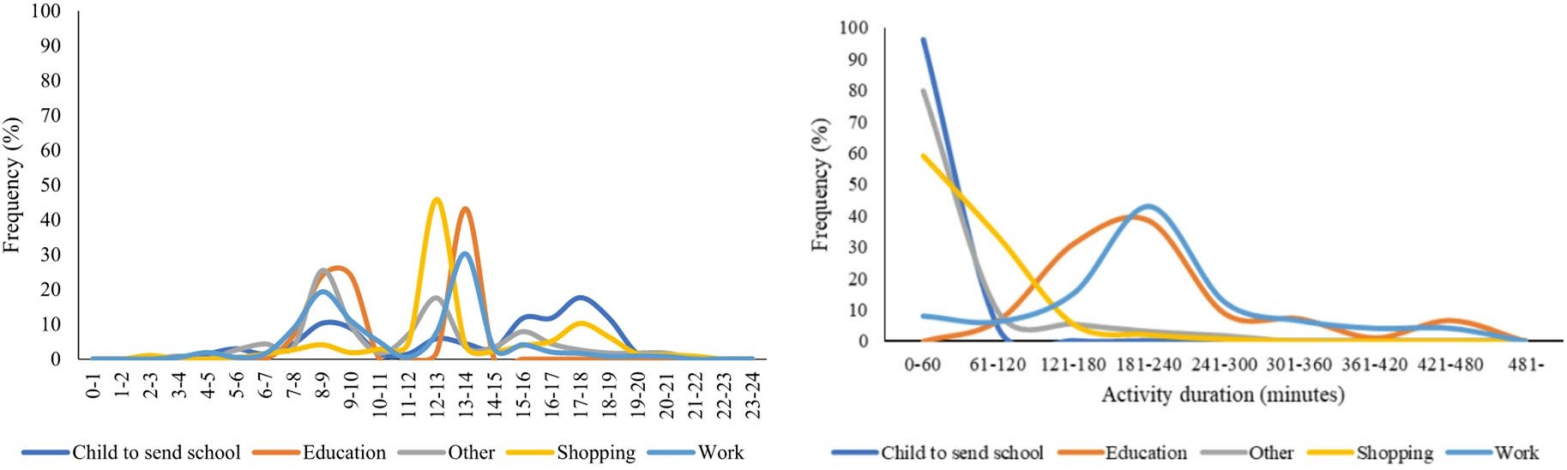
Distribution of start time (Left) and duration (Right) for each type of activity.

Finally, we determined the schedule of daily activities and travel behaviors for each individual by analyzing the distributions of activity start times and durations. The ADS results were aggregated and summarized as relative frequency distributions (Fig. 5 (left)). Regarding work and school activities, they mainly occurred during morning peak (7:00–9:00 AM) and midday (12:00–2:00 PM) times. In contrast, shopping activities were most frequent during midday (12:00–1:00 PM), followed by an evening peak (4:00–7:00 PM).

The duration of activities (which likely include work breaks) associated with 42.9% of the workers was 181–240 min(Fig. 5 (right); 80%–90% of individuals engaged in ‟other” activities, including ‟child drop/pick” activities with durations of 0–60 min; Similarly, the durations of shopping activities associated with 58.9% of the workers were 0–60 min; those associated with 33.1% of the workers were 61–120 min. Based on these distributions, we can assign appropriate activity durations when generating the daily activity schedule of an individual to obtain a detailed and realistic representation of their activity patterns.

#### Traffic demand forecating using ABM

Using the synthetic population data and the associated parameters, we estimated the daily traffic demand based on the probabilities of choices of activities and travel behaviors. Using ABM, travel behaviors and activities are formulated according to the following processes: (1) activity pattern generation, (2) destination and mode choices, and (3) scheduling of activities.

1. Activity pattern generation

The following models are used in this process:

1. An outbound-stop model for calculating the number of stops between home and the first main activity (work or school).
2. A subtour model for predicting the existence or absence of a main location-based subtour (a series of activities starting and ending at the main destination is referred to as ‟subtour” (work–leisure–work).
3. An inbound-stop model for calculating the number of stops on the way back to the starting point.
4. A multinomial logit model for each secondary activity for determining the type of activity during tours. Each individual has a desired activity pattern that includes the number of activities and their order within a tour, once the number of tours and stops during a tour has been decided. However, these patterns do not contain information about the destinations of each activity and times of day (scheduling of each individual).

(2) Destination and mode choices.

The choice of activity destination and mode choices for each trip was based on probability. For mode choice, we employed a logit model based on the utility of each mode choice between zones, as described in Eq. (2).

2 $$P(m\left| {ij} \right.)\; = \;\frac{{\exp (\mathop V\nolimits_{ijm} )}}{{\sum\nolimits_{k} {\exp (\mathop V\nolimits_{ijk} )} }}$$

where, $\mathop V\nolimits_{ijm}$: The utility of mode m on OD pair zone i to zone j , k:all alternative mode.

The log sum variable or expected maximal utility of mode choice is then Eq. (3):

3 $$\mathop {EMU}\nolimits_{ij} \; = \;\ln \{ \sum\limits_{m} {[\exp \left( {\mathop V\nolimits_{ijm} /\phi } \right)]} \}$$

Several factors, including travel distance and travel time, influence each trip’s modal choice. Mode is nested into a destination by integrating the expected maximum utility (EMU) of mode choice into the destination choice utility, as indicated in Eq. (4). This information affects the destination choice and the level of service on all modes. In this formulation, mode choice is nested within destination choice, allowing accessibility by all available modes to influence destination attractiveness.

4 $$V(j\left| i \right.)\; = \;\ln (\mathop A\nolimits_{j} )\,\, + \,\,\theta \,\, \cdot \,\,\mathop {EMU}\nolimits_{ij} \; + \;\mathop \lambda \nolimits_{i} \; + \;\mathop \lambda \nolimits_{ij}$$

where, $\mathop A\nolimits_{j}$: Attraction of zone j,$\mathop \lambda \nolimits_{j}$: Shadow price of destination j,$\mathop \lambda \nolimits_{ij}$: Shadow price of OD pair ij.

For secondary activities, destinations are selected in consideration of the positional relationship of home, workplace (school), etc., Eq. (5):

5 $$\begin{aligned} V(j\left| i \right.) = & \,\ln (\mathop A\nolimits_{j} )\,\, + \,\,\,\alpha \;\,(\theta \,\, \cdot \,\,\mathop {EMU}\nolimits_{ij} \; + \;\mathop \lambda \nolimits_{j} + \;\mathop \lambda \nolimits_{ij} ) \\ & \, + \,\,\,\beta \;\,(\theta \,\, \cdot \,\,\mathop {EMU}\nolimits_{ij} \; + \;\mathop \lambda \nolimits_{j} + \;\mathop \lambda \nolimits_{ij} ) \\ \end{aligned}$$

where, $\alpha$: weight of trip origin, $\beta$: weight of trip destination.

Finally, the selection of destination j under the condition of starting at origin i is described in Eq. (6).

6 $$P(j\left| i \right.)\; = \;\frac{\exp (V(j\left| i \right.))}{{\sum\nolimits_{k} {\exp (V(k\left| i \right.))} }}$$

This approach does not explicitly model destination choice and mode choice as a fully simultaneous or jointly optimized decision process. Instead, at this stage of the model, destination choice and mode choice are treated sequentially, whereby individuals are assumed to select the travel mode that maximizes utility for a given origin–destination pair. Importantly, this preliminary choice process does not explicitly incorporate individual daily time constraints or activity time budgets. Consequently, individuals may select the most preferred mode in terms of utility without fully considering whether the resulting travel time is feasible within their overall daily schedule. This simplification may lead to unrealistically long travel times and potential violations of individual time budgets at the pre-scheduling stage. Nevertheless, this formulation provides an initial approximation of preferred destination and mode combinations based on perceived utility. All destination and mode choices at this stage are computed using the estimated behavioral parameters obtained in Sect. “Identification of changes in activities and estimation of the necessary parameters” (b), thereby ensuring consistency in preference representation across model components.

(3) Tour scheduling and generation.

Rule-based scheduling is used to generate a consistent daily plan based on previously selected activities and tours. This process is performed in two steps:

- Step 1: Determine the start time and duration of each activity.

This is achieved using the probability distribution of each type of activity obtained from ADS (Fig. 5).

- Step 2. Setting time constraints for activity and trip.

Using this distribution, each activity is assigned a duration randomly. Next, the final schedule is established by ensuring that the time constraints are met. If not, the calculation starts from the activity pattern, destination, and TM (Traffic Mode). Finally, individual tour data are generated. These data are then divided into individual trip data, and an OD matrix is constructed for each mode.

#### Traffic assignment

Using the OD matrices by transportation mode generated in step (c) and the road network data, traffic assignment was performed in a manner similar to the four-step model. The resulting OD-based travel times and shortest path distances for each transportation mode were used as service level indicators, which were then incorporated into the next iteration of traffic demand forecasting. This iterative process was repeated until the difference in assigned traffic volumes between successive iterations converged to within 1% or until 100 iterations were completed. The final traffic demand forecasting for the entire urban area was based on the results of this iterative procedure.

#### Representation of flood impacts on the model

To evaluate the impact of road disruptions and speed reductions caused by floods, we categorized road segments according to their typical travel speeds. Then, we formulated a function describing the relationship between speed reduction and flood depth. This function was then applied to the flood depth of each road segment to evaluate the speed reductions and disruptions specific to each segment.

To formulate this function, we used taxi probe speed data of critical road segments (Bypass and Main Road) in Ubon Ratchathani since September 2019 provided by the Intelligent Traffic Information Center (iTIC) Foundation, Using these data, we compared travel speeds during usual conditions and flood conditions occurred between 12:00 p.m. and 6:00 p.m. from September 10 to September 17, 2019. Data outside these periods was considered usual-conditions data.

**Fig. 6:**
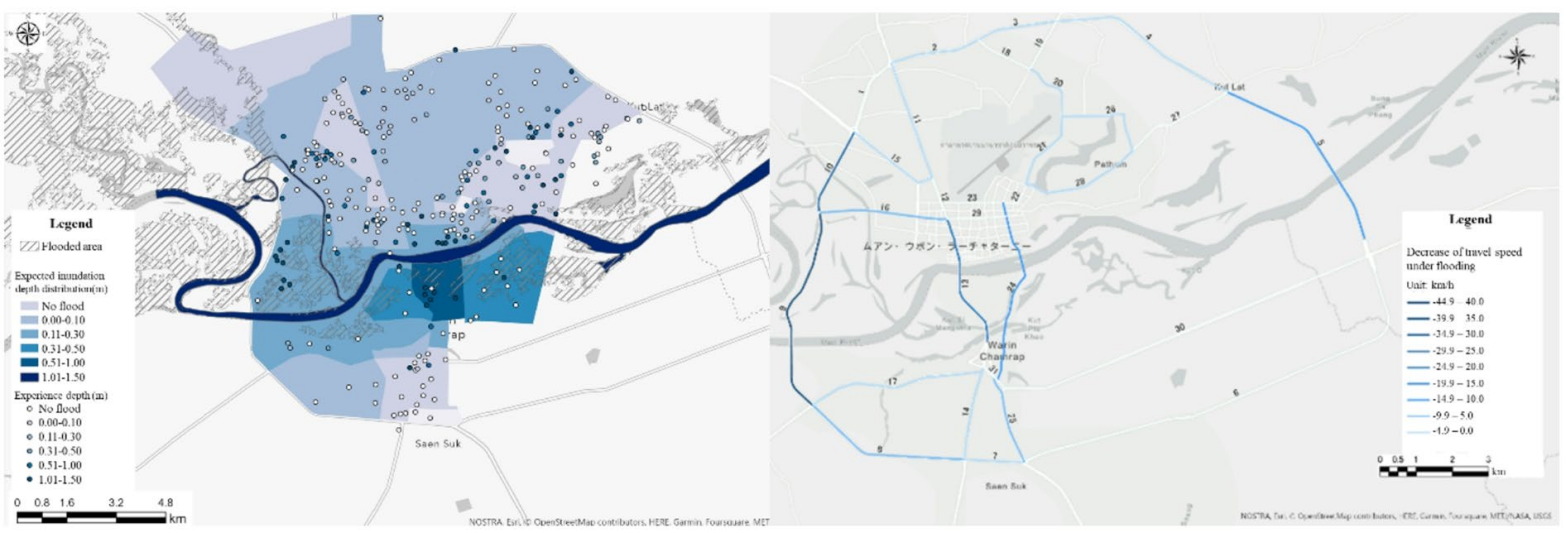
Inundation depth (Left) and decrease in travel speed of road segments (Right).

Additionally, we collected 300 samples of flood-depth data by conducting a questionnaire survey. We plotted these data using Geographic Information Systems and estimated the distribution of flood depths throughout the city using the Kriging method (Fig. 6 (left)). The estimated inundation depths were validated by comparison with flood extent data published by Sentinel Asia based on satellite imagery during flood events, confirming that areas with greater inundation depths generally correspond to zones with more severe flood impacts. Similar spatial patterns were also observed when compared with flood area data from the local Irrigation Office. Based on these comparisons, the inundation depth data were used in the analysis.

To evaluate the reduction in travel speed due to flooding, we compared travel speeds for each road segment during usual and flooding conditions (Fig. 6 (right)). The comparison showed that the travel speeds in the Western and Eastern Bypasses as well as two bridges in the city center (south of the Central Business District) were significantly affected by flooding. Specifically, the speeds in the Western and Eastern Bypasses decreased by − 40 km/h and − 12 km/h, respectively. Additionally, the two central bridges were flooded, disrupting road segments.

Furthermore, the estimated distribution of flood depths was combined with travel speed data of the road network. The reduction in travel speed for various flood depths was assessed for each speed category (21–30, 31–40, 41–50, 51–60, and 61–70 km/h), and a function describing the relationship between speed reduction and flood depth was formulated.

**Fig. 7:**
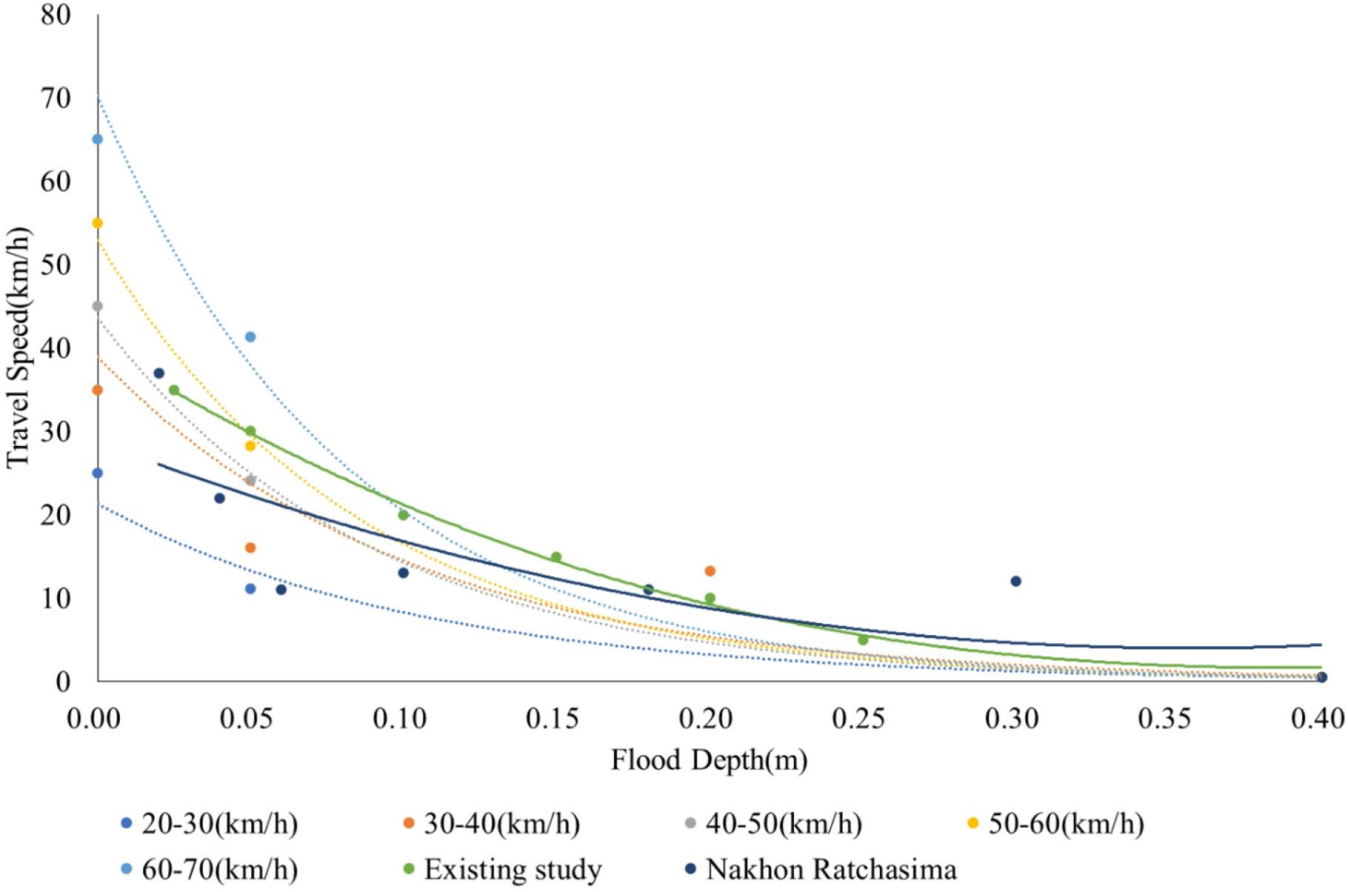
Function of decrease in travel speed and inundation depth.

The elevated road segments were excluded from the analysis. The flood-depth threshold beyond which crossing becomes impossible was set to 0.4 m according to the past studies^43,44^. The results are presented in Fig. 7. We observe that, for small flood depths (less than 5 cm), the reduction in travel speed is strongly influenced by the initial travel speed. The applicability of this function to real-world conditions was examined by comparing it with a speed–depth relationship estimated from a travel speed survey conducted in Nakhon Ratchasima, Thailand. The dataset was derived from approximately one hour of video footage recorded at a flooded roadway section, during which vehicle movements under varying inundation depths were continuously observed. In total, 208 vehicles were identified and analyzed based on image-based trajectory and speed extraction.The estimated functional form is presented in Eq. (7), and the comparison indicates good agreement between the two functions, with a coefficient of determination (R^2^) of 0.6073.

7 $$y = 194.67\mathop {\rm X}\nolimits^{2} - 138.73{\rm X} + \;\;28.727$$

where, y: travel speed (km/h), x: inundation depth.

In this study, flooding conditions are represented using static inundation depths based on estimated flood depth data.This simplification is justified by the hydrological characteristics of the study area, where flood events typically persist for extended periods and water levels remain high for a substantial duration before receding. Under this assumption, it provides a practical approximation of the flood exposure relevant to effects of activities and travel behaviors under flooding situations.

### Riverine flood damage in study area and responses

**Fig. 8:**
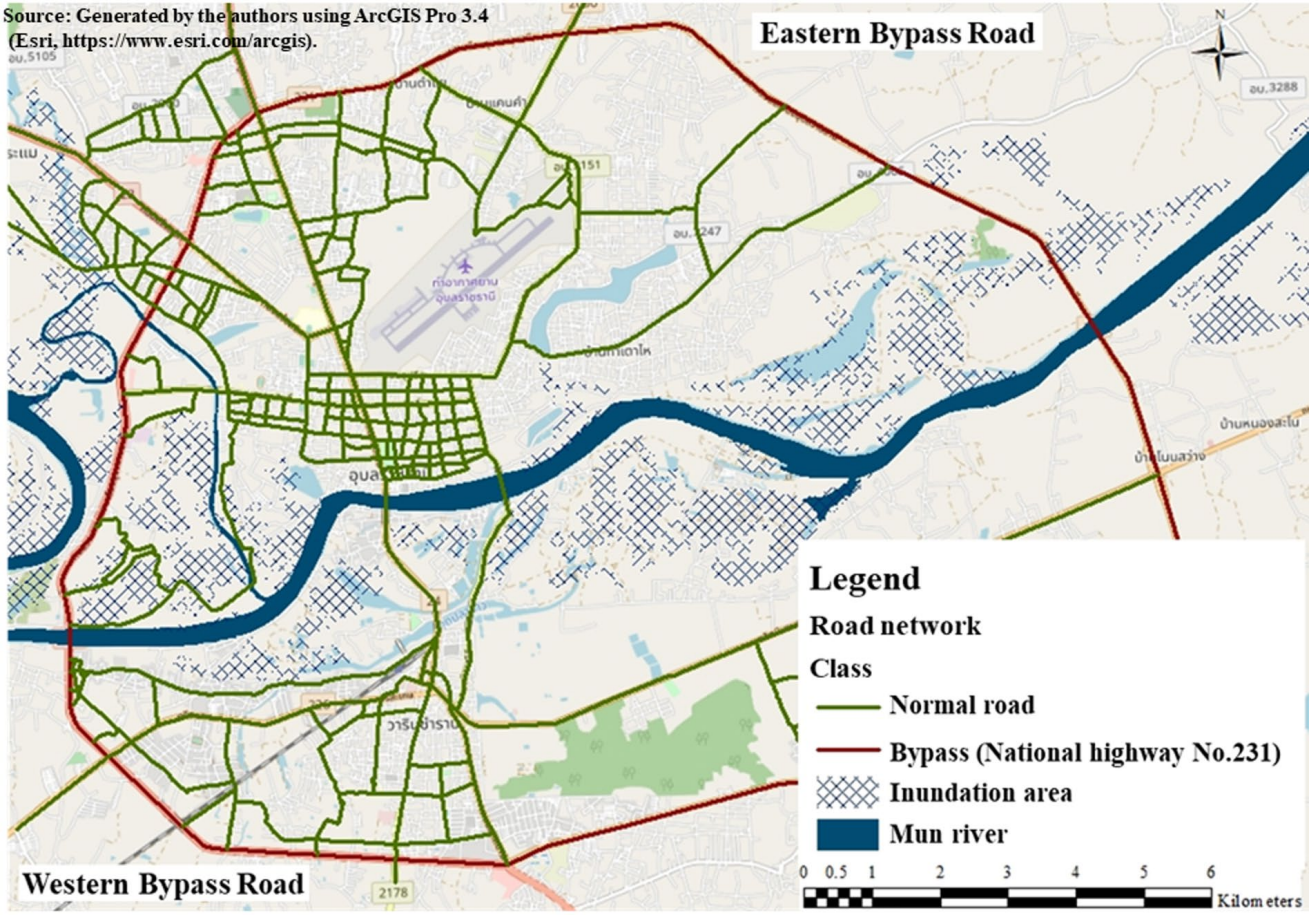
Case study: Ubon Ratchathani, Thailand.

As a case study, we selected Ubon Ratchathani, a city in northeastern Thailand. The city experiences a tropical monsoon climate with a distinct rainy season (May to October) during which heavy rainfall frequently results in floods. The river Mun, which is a major tributary of the river Mekong, flows through the city and causes significant floods. The river Mun divides the urban area into two distinct regions (northern and southern, Fig. 8).

In recent decades, Ubon Ratchathani has been rapidly urbanized. The conversion of natural areas into urban zones has resulted in a reduction in the natural absorption capacity of the land, making it susceptible to flooding during heavy rainfall and reducing the effectiveness of natural measures against flooding. The city has suffered significant damage caused by riverine floods, which have been intensified by climate change and extreme weather conditions.

Historical records have revealed severe floods in 1938, 1950, 1978, 1998–2002, 2019, and 2022, indicating the city’s long-standing vulnerability, which is attributed to inadequate flood protection and adaptation measures. The 2002 flood caused severe damage to buildings, infrastructure, and road segments near the river Mun, including four important bridges. The property damage exceeded 1 billion THB^45^.

Although flood-control measures, such as floodwalls, are currently underway, severe damage may occur during riverine floods. Residents have responded by changing their behaviors. Therefore, it is necessary to implement adaptation measures to maintain a certain level of urban mobility.

### Validation of traffic demand forecasting under usual situation by ABM

**Fig. 9:**
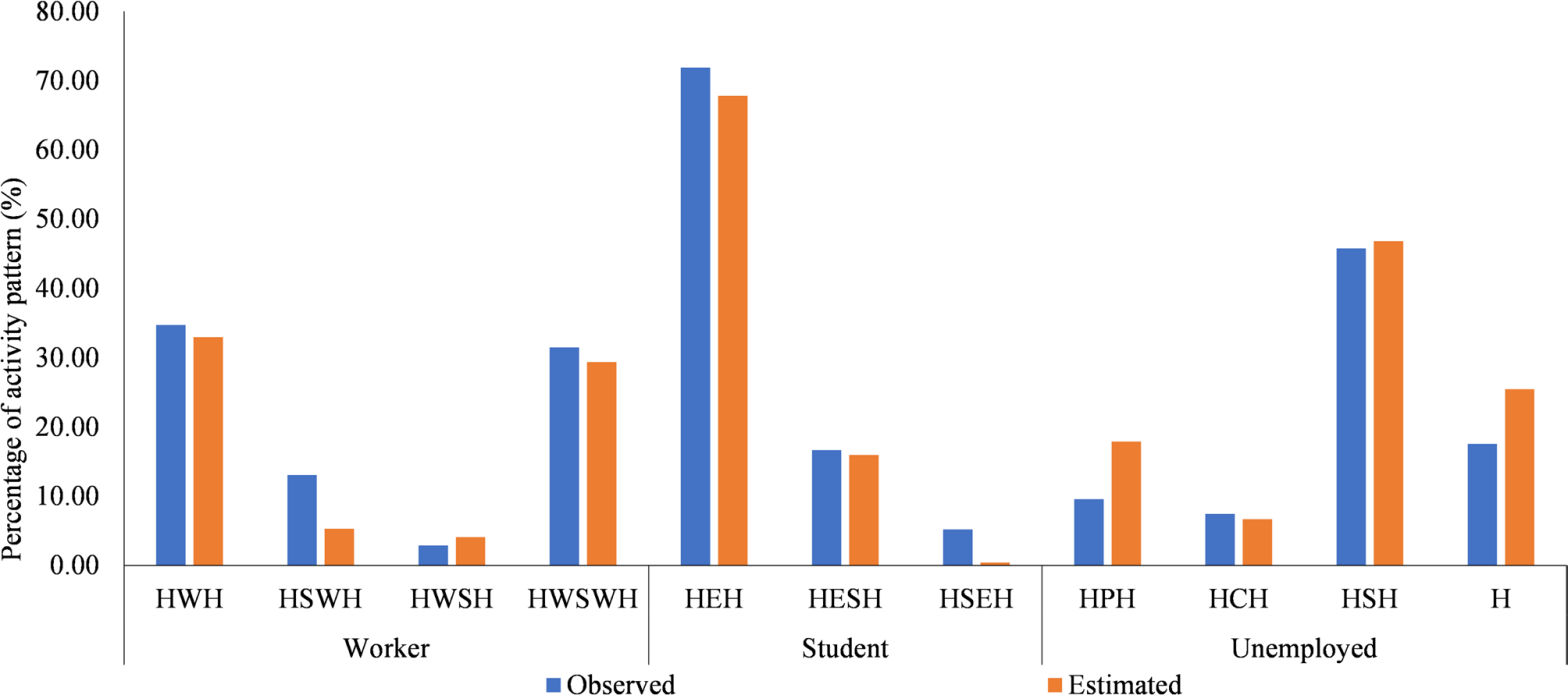
Comparison of activity patterns between estimated data and survey data.

Based on the estimated parameters and the synthetic population, travel demand was forecast under usual conditions using ABM. The predicted results were validated by comparing the activity patterns of three distinct groups (workers, students, and nonworkers) obtained using ABM with the ADS data, (Fig. 9). The deviation was − 5 ~ 8%, which is attributed to the overestimation of the number of people staying at home (for example, nonworkers usually stay at home longer than those in other categories). Under the usual conditions, most activity patterns forecast using ABM closely match the survey results.

**Fig. 10:**
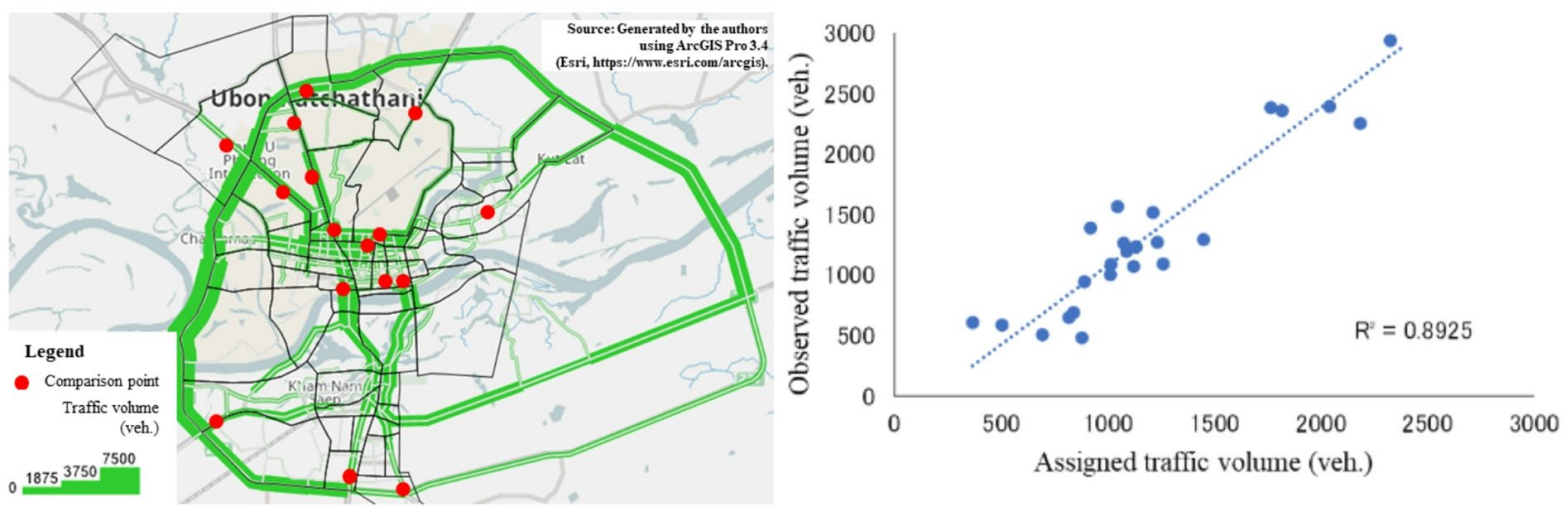
Observed and estimated traffic volume during peak hour (Left: Traffic Asignment; Right: Correlation Analysis).

The data obtained using ABM during the hourly evening peak (16:30–17:30) were extracted, and the traffic volume was assigned via user equilibrium assignment (Fig. 9). The traffic volume was assessed by comparing the observed traffic volumes at 16 locations (Fig. 10 (Left)). The traffic assignment results were compared with the hourly traffic volumes recorded during the same period at the 16 locations in both directions (shown in red in Fig. 10 (Left)). These traffic volume survey data at 16 locations were conducted as a supplementary survey to a person-trip survey of Ubon Ratchathani City in 2015. The survey locations included major arterial roads, including Ubon Ratchathani City’s main bypass highways, Routes 231 and 24, as well as urban roads, including the city’s CBD. These data were collected at locations that captured the city’s main traffic conditions. The calculated R^2^ was 0.8925, indicating that ABM can predict the actual traffic conditions (Fig. 10 (Right)). In addition, traffic volume surveys were also conducted at the same locations during the morning peak (7:30–8:30) and afternoon off-peak (11:30–12:30) periods. The R^2^ values were relatively good, exceeding 0.75, for both periods.

The travel demand results estimated under usual (non-flooded) conditions using ABM were compared with the TM-share results obtained from PTS (Table 7). Good agreement is observed between the ABM and survey results. The maximum deviation observed was 3.52%. From these results, it is possible to reproduce the traffic demand forecasting by the ABM accurately.

**Table 7:** Comparison of modal share by ABM and PTS.

Mode	ABM (%)	PTS (%)	Difference (%)
Car	52.18	55.70	− 3.52
Motorcycle	46.49	43.00	3.49
Public transport	0.88	1.10	− 0.22
Walk	0.45	0.20	0.25

The above results show that ABM is a robust and practical tool for traffic demand forecasting. It captures activity patterns and overall travel behaviors by incorporating the diverse behavioral characteristics of different types of populations. Therefore, it is well-suited for reproducing the existing conditions and assessing potential changes resulting from the implementation of transport measures or policy interventions.

## Adaptation measures in the transport sector and scenarios

### Adaptation measures in the transport sector

To select the adaptation measures that ensure urban mobility during floods, this study reviewed the measures recommended by the Japan International Cooperation Agency (Climate-FIT (Adaptation) tool), Japan Climate Change Adaptation Plan, reports from the European Environment Agency and the Asian Development Bank as well as existing studies (Table 8)^46–53^.

**Table 8:** Adaptation measures in the transport sector.

Classification	Measures
Soft	Improvement of Climatic prediction
	Forecasting of extreme weather conditions
	Regulations of Information related to extreme weather
	The use of hazard maps
Hard	Elevation of Road segments
	Implementing of emergency road segments
	The use of drainage asphalt

Among the adaptation measures, this study evaluated road elevation because it has already been implemented in many cities (Fukuchiyama, Japan, and Phnom Penh, Cambodia).

### Prioritization of adaptation measures

Prioritization methods for handling natural hazards, including floods, have been reported in many previous studies from the perspective of vulnerability. These methods include multiple-criteria decision analysis, serviceability analysis (including accessibility), and network connectivity assessment^54–56^. Prioritization indexes based on spatiotemporal distances or spatial proximity have been employed to mitigate the adverse effects of disaster. However, many of these methods only considered the severity of disasters without including the probability of their occurrence.

Historically, accessibility has been defined as “the potential opportunities for interaction” and “the ease of reaching land use given the transport system”^57,58^. In this study, accessibility is defined as the ease of performing an activity at the desired time and location under usual or flood conditions; it indicates the proportion of facilities that can be reached within 15 min from the center of each traffic analysis zone. For the case study, we created road network data and identified zone centers (55 in total) using Points of Interest (POI), including 134 workplaces, 34 schools, 73 hospitals, 192 shopping centers/markets, and 26 parks.

Based on these data, we initially evaluated the number of accessible facilities under usual conditions using Network Analysis in ArcGIS Pro. Then, we overlaid the flood-depth data onto the road network to determine the decrease in accessibility during floods. Each road segment was assigned several attributes, including reduced travel speed or road closures based on the level of flood depth, to represent the reduced functionality of the road network. In this way, we estimated the reduction in citywide accessibility caused by flooding by comparing the results with those obtained under usual conditions. Additionally, we conducted an analysis assuming that road segments affected by flooding could still function similarly to usual conditions. We utilized the flood-affected network to calculate the proportion of accessible facilities within a 15-min threshold for the five facility types in each zone using Eq. (7). The overall city accessibility was calculated using Eq. (8).

**Fig. 11:**
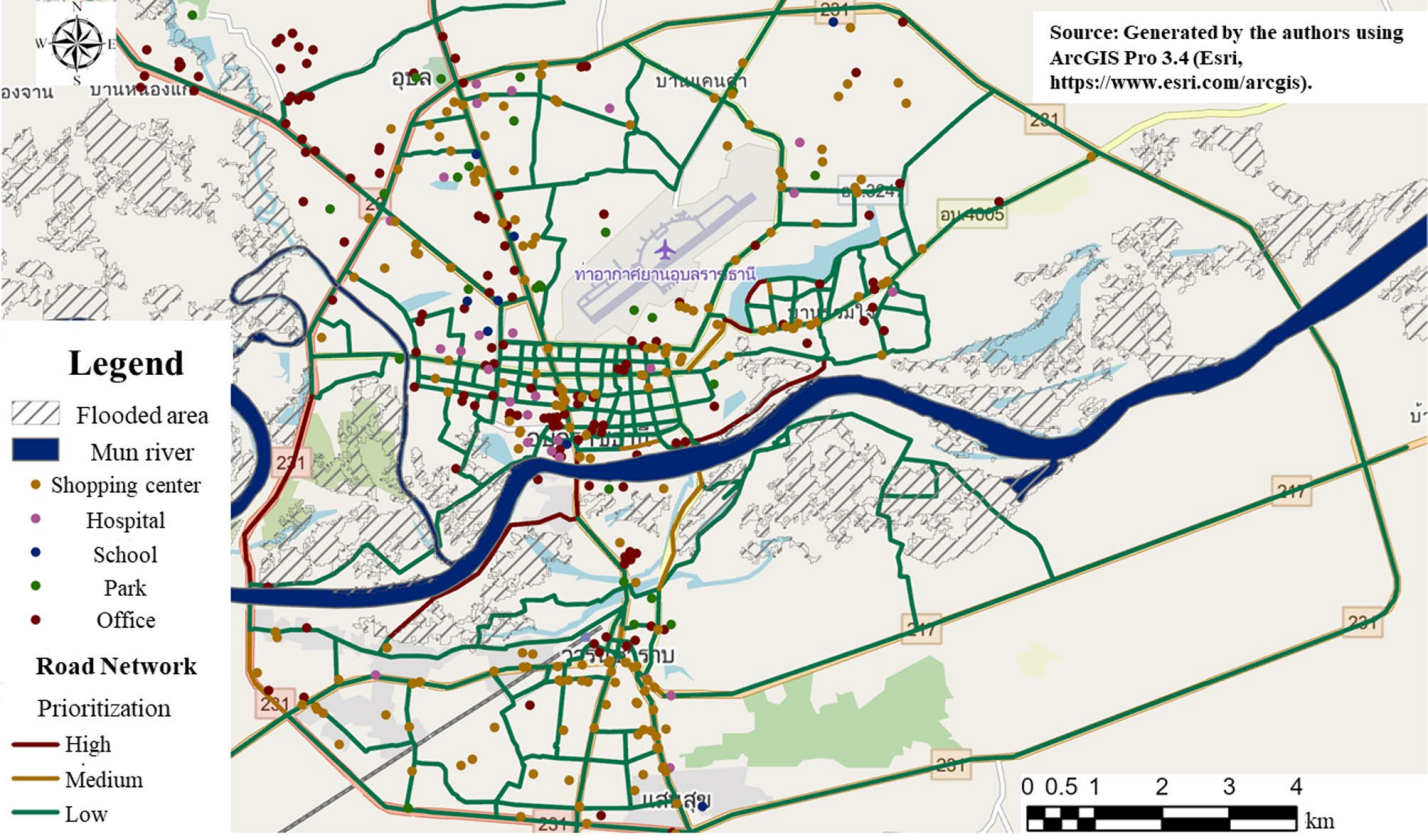
Prioritization result for road segments.

By comparing accessibility during floods, we evaluated the impact of 104 affected road segments, assuming that these segments remained operational during floods. Using Eq. (9), we set the following prioritization thresholds: a citywide accessibility improvement of 5% or more was classified as “Medium,” whereas an improvement of 10% or more was classified as “High.” The results are presented in Fig. 11. Among the flood-affected road segments, eight segments (9.11 km) were classified as having a medium priority (shown in yellow), and seven segments (11.47 km) were classified as having a high priority (shown in red) These results were used to estimate the potential improvements of raising the elevation of the selected road segments in each scenario.

7 $$\mathop {ACC}\nolimits_{i} = \frac{{\sum\limits_{f = 1}^{n} {\mathop {ACC}\nolimits_{if} } }}{{\mathop X\nolimits_{f} }}$$

where, $\mathop {ACC}\nolimits_{i}$: Accessibility of zone i, $\mathop {ACC}\nolimits_{if}$: Accessibility of facility f in zone i, n: Number of facility type, $\mathop X\nolimits_{f}$: Number of facility of type f

8 $$TA = \sum\limits_{i = 1}^{i} {\mathop {ACC}\nolimits_{i} }$$

where,$TA$: Accessibility of the whole city

9 $$\mathop {TAR}\nolimits^{with} = \frac{{\mathop {TA}\nolimits^{with} - \mathop {TA}\nolimits^{without} }}{{\mathop {TA}\nolimits^{usual} - \mathop {TA}\nolimits^{without} }}$$

where, $\mathop {TAR}\nolimits^{with}$: Relative accessibility evaluation after road elevation, $\mathop {TA}\nolimits^{with}$: Accessibility with implemented countermeasures, $\mathop {TA}\nolimits^{without}$: Accessibility without countermeasures, $\mathop {TA}\nolimits^{usual}$: Accessibility under unusual conditions.

### Scenario setting and evaluation criteria

Multiple scenarios were set based on the prioritized road segments identified in the previous section (Table 9). Scenario A corresponds to the baseline case (no adaptation measures); Scenario B corresponds to elevated medium-priority road segments; Scenario C corresponds to elevated high-priority road segments; Scenario D corresponds to elevated medium- and high-priority road segments. This study compared Scenarios B, C, and D against Scenario A to evaluate the effectiveness of the road elevation measures.

**Table 9:** Scenario setting.

Scenario	Setting of pattern
A	Without adaptation measures
B	Elevation of medium-priority road segments
C	Elevation of high-priority road segments
D	Elevation of medium- and high-priority road segments

This study assessed these scenarios by comparing them with the baseline scenario (Scenario A) to estimate the impact of riverine floods on activity patterns, traffic assignment, travel time to workplace, and benefits of travel time reduction. Specifically, this study analyzed the activity patterns by categorizing individuals into the three groups defined in Sect. “Traffic Demand forecasting by activity-based model” (b) to assess the impact of road elevation on individual ability to maintain their usual activities under flood conditions.

Additionally, this study investigated the degree of reducing the adverse effects of floods to ensure urban mobility; for this purpose, this study analyzed the travel times to workplaces and the traffic assignment results. Finally, a Cost–Benefit Analysis (CBA) was conducted to evaluate the Benefit–Cost Ratio (BCR) for each scenario to assess the economic feasibility of implementing arterial road elevation as an adaptation measure.

## Results of the case study

### Activity pattern

ABM predicted the total travel demand during a day for various scenarios. Based on the results, the activity patterns were categorized into three groups (workers, students, and unworkers) aligned with the classification employed in ADS. Table 10 presents a comparison of the activity patterns.

**Table 10:** Comparison of activity pattern in each scenario.

Classification	Activity pattern	Usual	A	B	C	D
Worker	HWH	50,098	47,555	49,779	50,688	52,693
	HWOH	283	29	38	29	41
	HWSH	316	46	48	56	58
	HWSWH	29,828	35,102	31,441	30,172	30,007
Student	HEH	31,665	32,115	33,688	34,562	35,459
	HOEH	2,460	1,912	2,006	2,005	2,410
	HEOH	6,298	4,844	5,081	5,327	6,152
Nonworker	HOH	38,174	27,957	29,326	30,009	29,424
	HCH	4,244	3,936	3,931	3,944	3,096
	HSH	21,058	1,615	17,117	18,371	15,963
	H	54,759	70,832	63,857	62,988	61,601

The finding revealed that during floods, the proportion of people staying at home all day (activity pattern: H) increases by approximately 15% (from 54,759 individuals under usual conditions to 70,832 during floods), which is consistent with the survey results. Conversely, the proportion of workers engaging in shopping activities- either during their commute home or during work breaks (Activity Pattern: HWSWH)- increased by about 10%. This indicates that workers often engage in activities typically performed by unworkers, as a result of the reduced mobility during floods.

However, by elevating arterial roads, the number of individuals staying at home decreases by approximately 13% compared with the baseline scenario (no adaptation measures). This is attributed to improved urban mobility, which allows people to perform their usual activities. Additionally, the number of workers that go shopping during their commute to home or make work breaks decreases by approximately 5%. This indicates that without road elevation, nonworkers face mobility challenges, leading workers to shop on their behalf. Once mobility is restored, nonworkers return to their usual activities.

### Travel time on trips to workplace

**Fig. 12:**
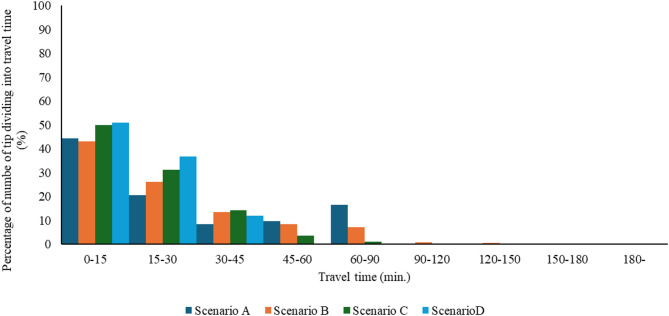
Percentage of trips to the workplace of travel time.

In order to understand the impact of flooding on mobility, we categorized each trip based on travel time and compared the proportions. Figure 12 illustrates the findings for work-related trips. Under usual conditions (without flooding), 95% of trips have a travel time of less than 15 min. However, during flooding events (without any countermeasures), if road elevation is not implemented, approximately 25% of trips experience a travel time of 50 min or more.

In contrast, when road elevation is introduced as a high-priority measure, the maximum travel time for all trips is reduced to 45 min. This outcome demonstrates that implementing road elevation significantly mitigates the effects of flooding on urban mobility. Therefore, the appropriate integration of road elevation is essential to ensure mobility and facilitate smooth transportation, even during flood events. A similar trend was observed for other trips as well.

### Traffic assignment

**Fig. 13:**
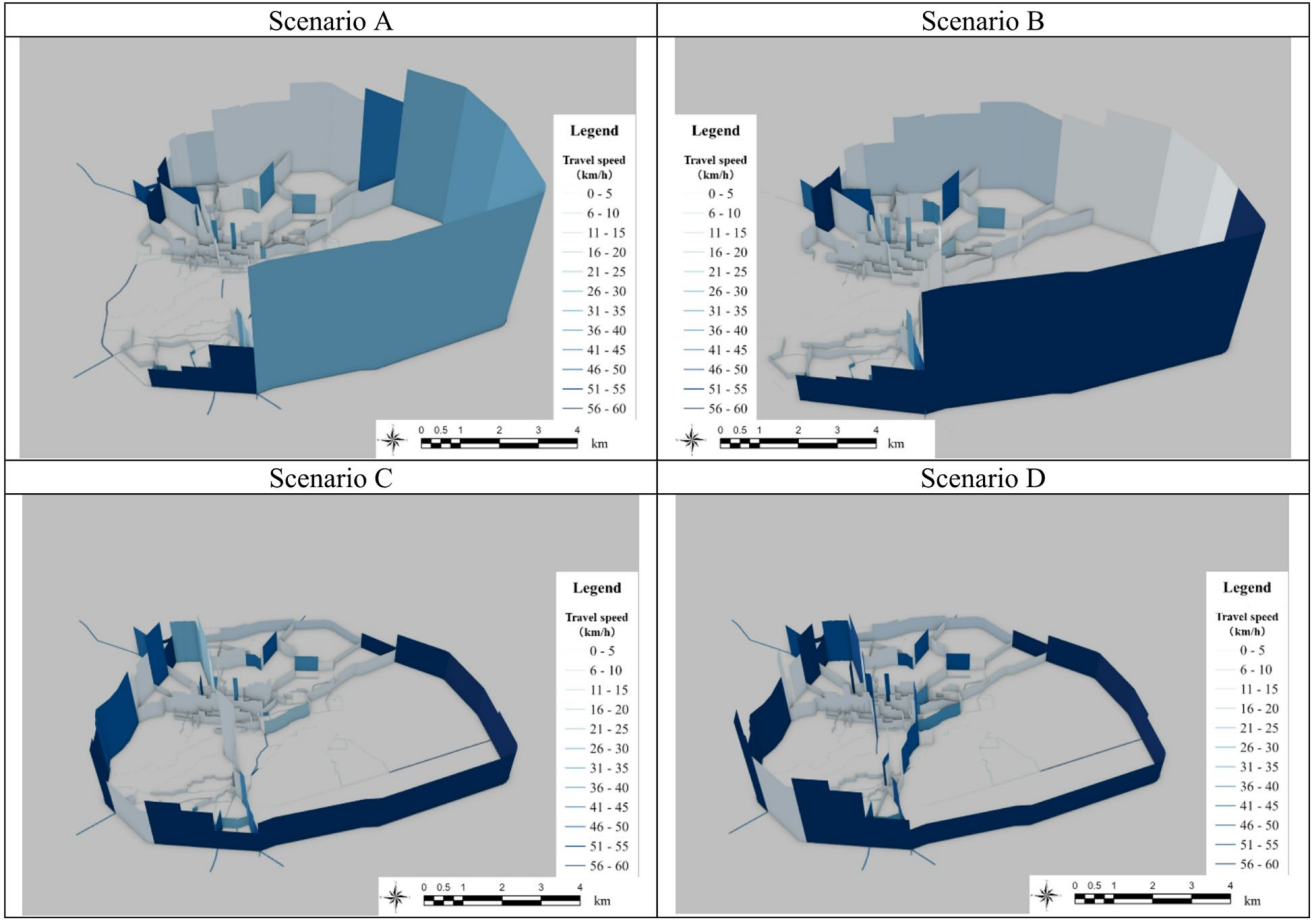
Results of traffic assignment each scenario.

The traffic assignment results for each scenario were obtained using ABM (Fig. 13), where the height of each road segment and the color intensity indicate the traffic volume and travel speed, respectively. These visualized results indicate the extent of detours under flooding conditions and the overall changes in traffic across the road network. By comparing different scenarios, these results can evaluate the effectiveness of arterial road elevation in reducing traffic disruptions caused by floods.

The results indicate that in Scenario A (no adaptation measures), 78,000 vehicles used the Eastern Bypass during the day, resulting in severe congestion and long travel times. In contrast, the arterial road elevation significantly reduced the number of detouring vehicles, resulting in improved traffic flow. In Scenarios B, C, and D, the bypass was decreased by approximately 54,000, 17,800, and 16,800 vehicles, respectively. These results demonstrate the significant impact of road elevation on reducing detours and improving urban mobility during floods.

Notably, the elevation of the Western Bypass and central bridge (left side of the study) was essential in reducing detours and ensuring continuous access to major road segments. Additionally, it increased travel speed of whole network, especially in the Western and Eastern Bypasses. These improvements indicate that critical infrastructure upgrades can substantially boost network resilience and lessen the negative impacts of flooding on urban mobility.

### Cost benefit analysis

This study evaluated the economic efficiency of the proposed scenarios by conducting CBA; based on the traffic assignment results for each scenario, this study compared the economic damage caused by floods with and without implementing the elevation of road measure. The CBA results showed a reduction in damage costs due to improved traffic conditions and reduced disruptions during floods, indicating the importance of adaptation measures. Our CBA evaluation followed the guidelines of the CBA Manual published by the Ministry of Land, Infrastructure, Transport, and Tourism of Japan. Additionally, we evaluated BCR for each scenario by dividing the estimated benefits by the implementation costs of road elevation (Table 11).

**Table 11:** Cost benefit analysis results.

	Cost of road elevation (mil-THB)	Effectiveness of disaster prevention (mil-THB)	CBR
Scenario B	508.1	959.6	1.89
Scenario C	708.3	1555.6	2.20
Scenario D	1216.4	1971.7	1.62

In this study, a social discount rate of 4% was applied over an evaluation period of 20 years. In calculating the annual benefits of each scenario, we assumed that a flood of similar intensity would occur once per year, lasting for one month during the rainy season, the remaining eleven months were assumed unaffected by the riverine floods. The specific calculation procedure followed the methodology of Tsumita et al. (2024); readers are referred to that study for additional technical details^59^. The effectiveness of disaster prevention (benefit), which indicates the total reduction in flood damage, for Scenarios B, C, and D was estimated at 959.6 mil-THB, 1,555.6 mil-THB, and 1,971.7 (mil-THB), respectively. In all scenarios, BCR > 1.00, indicating that the proposed road elevation is economically viable. The BCR values for Scenarios B, C, and D were 1.89, 2.20, and 1.62, respectively; in Scenario C, road elevation was implemented in the most critical road segments, achieving the highest economic efficiency.

## Conclusion

This study analyzed the impact of floods on urban mobility in Ubon Ratchathani, Thailand, a city that experiences prolonged riverine floods annually. Initially, based on the ADS of the study area, this study investigated daily activities and travel behaviors under usual and flood conditions. Based on the survey results, this study evaluated the logit model parameters to explain tour frequency choice, activity choice (number of stops), and mode choice under each condition. These parameters were then used to develop an ABM.

Based on ABM, we selected the elevation of arterial roads as an adaptation measure in the transportation sector and evaluated its impact on urban mobility by assessing its effectiveness in critical road segments to select by accessibility indexes. The survey results showed that floods significantly affect activity patterns, particularly among workers. Compared with the usual conditions (31.5%), the proportion of workers engaging in shopping activities during work breaks or on their way home under flood conditions to 37.0%. Using ABM, these nuanced changes in individuals’ behavior can be represented at a disaggregated level, enabling a more accurate evaluation of the effectiveness of the adaptation measures. Based on these findings, this study developed an ABM to estimate the changes in travel demand caused by floods.

The elevation of arterial roads was chosen after reviewing various adaptation measures in the transportation sector. Using accessibility index, priority road sections prone to inundation or disconnection were identified and incorporated into scenarios. Then ABM was employed to estimate traffic demand in each scenario, the evaluations were based on activity patterns, trip time and assigned traffic volumes and BCR. The results showed that implementing road elevation effectively preserves urban mobility, allowing the activity patterns during floods to revert to those in usual conditions. Additionally, the traffic assignment results showed that prioritized road segments for reducing the number of detouring vehicles by approximately 24,000, 60,200, and 61,200 in Scenarios B, C, and D, respectively.

Furthermore, we conducted CBA to assess the economic feasibility of road elevation. The results showed that BCR exceeded 1.00 in all scenarios, confirming the economic viability of road elevation. Moreover, prioritizing high-priority road segments significantly improved efficiency and benefits. This study demonstrated the importance of resilient transportation networks and adaptive measures in sustaining urban mobility and activities during floods.

By leveraging the strengths of ABM to analyze behaviorally responsive and activity-based travel demand and integrating accessibility indicators and economic assessments, the findings demonstrate that the countermeasure effectively mitigates the disruptions caused by flooding, allowing individuals to maintain their usual travel behaviors and activities. This underscores the importance of proactive adaptation strategies in urban planning, ensuring that mobility remains viable even under extreme conditions. Enhancing transportation resilience not only minimizes economic losses but also supports social stability by preserving individual activities and traffic demands during disasters.

Moreover, the proposed methodology provides a practical framework for real-world applications, such as supporting urban flood risk management and informing transportation and land-use planning decisions. By quantitatively linking behavioral responses, accessibility changes, and economic impacts, the framework enables policymakers to evaluate and prioritize adaptation measures based on their effectiveness in preserving mobility and activity participation. Enhancing transportation resilience through such evidence-based planning not only reduces economic losses associated with disrupted travel demand but also contributes to social stability by sustaining individual activities and traffic flows during disaster situations.

At the same time, this study highlights the transferability of the proposed modeling framework beyond the case study area. With appropriate adjustments to data inputs, model calibration procedures, and context-specific behavioral assumptions, the framework can be applied to other regions with differing urban structures and hazard characteristics. While regional variations in travel behavior, data availability, and flood impacts must be carefully considered, the overall approach offers a flexible and scalable tool for analyzing behavioral responses to flooding across diverse urban contexts^60^.

Although this study focused on the elevation of arterial roads as an adaptation measure in the transport sector, further research is required to investigate alternative adaptation measures to ensure a more comprehensive approach to maintaining urban mobility. In addition, while the elevation of arterial roads can effectively preserve urban mobility during flood events, it may alter local drainage patterns and potentially generate new flood risks. Therefore, future studies should consider combining arterial road elevation with drainage improvements in order to mitigate possible secondary flooding effects.

Furthermore, this study has several limitations that should be addressed in future research. The flood-depth information used in this study is based on self-reported survey data, which may be subject to recall bias, differences in perception among respondents, and spatial uncertainty in reported inundation levels. In future studies, it will be necessary to conduct sensitivity analyses by systematically varying flood-depth levels to examine how changes in assumed inundation depths may influence the analytical results and conclusions.

Also, future research should explicitly address intra-household dependence in behavioral modeling. In particular, the use of cluster-robust standard errors at the household level, random-effects (mixed) logit models, or multilevel (hierarchical) modeling approaches would allow for a more rigorous treatment of unobserved heterogeneity and shared constraints among individuals within the same household. Applying such methods, in combination with larger and more detailed household-level datasets, would improve the robustness and interpretability of model estimates and provide deeper insights into individual and household decision-making processes under flood conditions.

## Data Availability

The datasets utilized and analyzed during the current study are available from the corresponding author on reasonable request.
